# HERVs and Epigenetic Regulators Transcriptional Expression After Chondrogenic Differentiation of Adipose Tissue-Derived Mesenchymal Stem Cells

**DOI:** 10.3390/cimb48010037

**Published:** 2025-12-26

**Authors:** Ilaria Galliano, Cristina Calvi, Stefano Gambarino, Alice Dato, Anna Pau, Maddalena Dini, Anna Clemente, Carlotta Castagnoli, Massimiliano Bergallo

**Affiliations:** 1 Department of Public Health and Pediatric Sciences, Medical School, University of Turin, 10124 Turin, Italy; ilaria.galliano@unito.it (I.G.);; 2 Laboratory of Specialistic Pediatry, Department of Pediatric Pathology and Care, Regina Margherita Children’s Hospital, Piazza Polonia 94, 10126 Turin, Italy; 3 BioMole Srl, Spin-Off University of Turin, 10135 Turin, Italy; 4 Skin Bank, Department of General and Specialized Surgery, University Hospital Città della Salute e della Scienza di Torino, 10126 Turin, Italy

**Keywords:** adipose-derived mesenchymal stem cells, human endogenous retroviruses, SETDB1, TRIM28, chondrogenic differentiation, pluripotency factors

## Abstract

Mesenchymal stem cells (MSCs) are multipotent cells capable of differentiating into various connective tissue cell types. Adipose tissue provides a rich source of MSCs (ADSCs), which can differentiate into osteoblasts, adipocytes, and chondroblasts. Pluripotency factors such as SOX2, NANOG, and OCT4 maintain MSC stemness, whereas human endogenous retroviruses (HERVs) and their epigenetic regulators TRIM28 and SETDB1 have been implicated in transcriptional regulation and cell fate decisions. This study investigated the transcriptional expression of HERV-H, -K, and -W, TRIM28, SETDB1, and pluripotency markers (NANOG, OCT4, SOX2) during chondrogenic differentiation of ADSCs using Real-Time PCR. Chondrogenesis was confirmed by aggrecan (ACAN) upregulation and aggrecan immunostaining. Although no statistically significant differences were observed for HERV-H, HERV-K, or HERV-W, HERV-K and HERV-W showed a trend toward decreased expression in differentiated cells, consistent with the overall shift in transcriptional profile during lineage commitment. TRIM28 expression was significantly reduced, while SETDB1 showed a decreasing trend. Among pluripotency markers, OCT4 was significantly downregulated, whereas NANOG and SOX2 remained stable. Correlation analyses revealed that in differentiated ADSCs, HERV-W expression correlated negatively with TRIM28 and positively with SETDB1, while no correlations were found for HERV-H or HERV-K. These findings suggest that specific HERV families and their epigenetic regulators may undergo coordinated modulation during chondrogenic differentiation, supporting a complex and family-specific interplay between retroelement regulation, pluripotency factors, and MSC lineage commitment.

## 1. Introduction

Mesenchymal stem cells (MSCs) are human multipotent cells derived from embryonic mesenchyme. They have the ability to develop into a variety of connective tissue types: bone, adipose tissue, cartilage, tendons, and muscle [1].

Various human organs and tissues store undifferentiated tissue-resident cells that support tissue repair and remodelling throughout life. Cells with MSC-like properties have been isolated from bone marrow, adipose tissue, skin, muscle, tendons, bone, brain, liver, kidneys, lungs, spleen, pancreas, thymus, synovial membrane, and the umbilical cord [2].

Adipose tissue has been shown to be a rich source of mesenchymal stem cells, comparable to those derived from bone marrow. Additionally, adipose-derived mesenchymal stem cells (ADSCs) exhibit similar characteristics in terms of differentiation potential, morphology, and phenotype to mesenchymal stem cells obtained from umbilical cord blood or bone marrow [3].

MSCs populations are heterogeneous and can be collected from many sources via different isolation, culture, and expansion approaches [1]. Due to variations in those methods, the Mesenchymal Stem Cell Committee of the International Society for Cellular Therapy (ISCT) has proposed 3 standardized criteria to determine if a cell qualifies as mesenchymal: adherence to plastic; multipotent differentiation potential to osteoblasts, adipocytes and chondroblasts under standard in vitro differentiating conditions and specific surface antigen (Ag) expression, such as CD105, CD90, and CD73 and lack expression (less than 2% population) of CD45, CD14, CD34 or CD11b, CD79a or CD19, and HLA class II [4].

MSCs are a type of adult stem cell known for their remarkable ability to self-renew and differentiate into multiple cell lineages. The transcription factors sex-determining region Y-box 2 (SOX-2), NANOG, and Octamer-binding Transcription factor 4 (OCT-4) play a crucial role in sustaining their pluripotency and self-renewal capabilities, ensuring their potential for regenerative applications and maintaining their undifferentiated state [5].

Approximately 8% of the human genome is derived from viral sequences known as human endogenous retroviruses (HERVs). These elements are remnants of retroviral infections that occurred in the germ line of ancestral primates over the past 100 million years. Over time, these sequences integrated into the genome and became stable components, serving as a boundary between the host’s own DNA and foreign genetic material [6].

HERVs are classified into three groups based on sequence similarity. Class I (gammaretroviruses) include HERV-E, HERV-F, HERV-T, HERV-V, ERV3 (HERV-R), ERV9, HERV-W, HERV-FRD, HERV-H, HERV-Fc, HERV-MER, and HERV-PABLB. Class II (betaretroviruses) including HERV-K family and Class III (spumaretroviruses) consist of the HERV-L family [7].

They can influence gene expression, with their integrated long terminal repeats (LTRs) functioning as alternative promoters. This activity can enhance the expression of nearby genes, potentially leading to the activation of oncogenes or the suppression of tumor-suppressor genes [5].

LTRs flank the three primary retroviral proviruses coding regions: group-specific antigen (*gag*), reverse transcriptase/polymerase (*pol*), and envelope (*env*), regulating their activity. Most viral DNA sequences in the human genome have been inactivated due to deletions or mutations that suppressed their open reading frames (ORFs). However, some HERVs retain complete ORFs, allowing them to produce viral proteins. Among the various HERV families, HERVK has the largest number of members with intact viral gene sequences [7].

HERV activation is regulated by Tripartite motif containing 28 protein (TRIM28) and SET domain bifurcated histone lysine methyltransferase 1 (SETDB1), which are part of the epigenetic mechanisms that organize the chromatin architecture in response to external stimuli [8]. HERVs are previously thought to coordinate with the host genome during mammalian evolution, and now they are considered as integral parts to form species and cell type-specific gene regulatory networks [9]. Among the various HERV families, HERV-H, HERV-K, and HERV-W are of particular interest in stem cell biology. HERV-H is highly expressed in pluripotent stem cells and contributes to the maintenance of transcriptional networks associated with self-renewal [9]. HERV-K retains the largest number of intact open reading frames among human ERVs and has been implicated in regulating cellular plasticity and stress responses [7]. HERV-W, by contrast, is known for its activity in immune and developmental pathways, and for shaping host gene regulation [6]. Previous studies from our group have also shown that these families are transcriptionally active in mesenchymal stem cells and undergo modulation under different physiological and pathological conditions, further supporting their relevance in MSC biology [5]. The research of ERVs in stem cell fate decision and differentiation has just been unraveled, and many questions remained to be answered. For this reason, in this study, we investigated the expression of HERVs, SETDB1, and TRIM28 in the chondrogenic differentiation of adipose-derived mesenchymal cells.

## 2. Materials and Methods

### 2.1. ADSCs Isolation and Expansion

Eighty lipoaspirate specimens were collected after written informed consent was obtained from all donors. Adipose tissue harvesting was performed using a low-pressure liposuction procedure with fenestrated blunt cannulas (Coleman technique) [10]. Immediately after collection, the lipoaspirates were centrifuged (at 3000 rpm, 3 min) to separate the adipose fraction. The fat layer was then washed twice with saline by centrifugation (1700 rpm, 10 min) and subsequently processed to isolate the stromal vascular fraction (SVF). Although 80 lipoaspirates were available, 10 independent samples were selected for ADSC expansion and chondrogenic differentiation, generating paired control and differentiated conditions for molecular analyses. Each sample was processed and analyzed individually, and no pooling of samples was performed.

### 2.2. Isolation of SVF from Adipose Tissue

SVF was obtained from fresh adipose tissue through enzymatic digestion with Collagenase NB6 (Serva Electrophoresis, Heidelberg, Germany; 0.3 U/mL). Digestion was carried out for 40 min at 37 °C under continuous mixing (tube rotator, Thermo Fisher Scientific, Waltham, MA, USA). Enzymatic activity was stopped by adding saline supplemented with 10% fetal bovine serum (FBS). The suspension was then centrifuged (1500 rpm, 10 min), and the resulting pellet was resuspended in saline. After sequential filtration through 100 µm and 70 µm strainers, cells were washed once more, centrifuged (1700 rpm, 5 min), and counted.

The mesenchymal stromal cell phenotype within the SVF was assessed immediately after isolation by flow cytometry. Surface markers profiling was performed on freshly isolated SVF and on SVF obtained after thawing and collagenase digestion. ADSCs were identified by positivity for CD105, CD44, CD73, and CD271, together with the lack of CD45 expression.

For immunostaining, fluorochrome-conjugated antibodies and matched isotype controls were used: CD105 PE (Invitrogen, Thermo Fisher Scientific, Waltham, MA, USA), CD73 FITC, CD44 FITC, CD45 PerCP, CD3 PerCP, CD271 APC, IgG1 PE, IgG1 APC, IgG2a PerCP (Miltenyi Biotec, San Jose, CA, USA), and IgG1 FITC (Immunostep, Salamanca, Spain). Approximately 10^5^ events per sample were acquired using CellQuest software, Version 5.1, and analyses were performed with FlowJo, Version 11 (Tree Star, Ashland, OR, USA).

Following SVF isolation, cells were plated in T25 flasks and cultured in Alpha Minimum Essential Medium (α-MEM; Sigma-Aldrich, St. Louis, MO, USA) supplemented with 10% human platelet lysate (HPL; provided by CPVE, Città della Salute e della Scienza, Torino, Italy), 2 mM L-glutamine, and 1% antibiotics (Gibco, Life Technologies, Carlsbad, CA, USA). After 24 h, non-adherent cells were removed by medium replacement. Thereafter, the medium was renewed every 48 h. When cultures reached ~85% confluence (typically 5–7 days), adherent cells were detached, reseeded, and expanded up to passage 3 for subsequent experiments.

### 2.3. Chondrogenic Differentiation

Expanded ADSCs were induced toward chondrogenic lineage using a commercial differentiation medium (StemMACS™ ChondroDiff Media; Miltenyi Biotec, Bergisch Gladbach, Germany). Chondrogenic induction was performed at passage 3 and maintained for 24 days, in accordance with the manufacturer’s instructions [11]. Undifferentiated control cells were cultured in α-MEM supplemented with 10% HPL under standard conditions.

For three-dimensional chondrogenic culture, 2.5 × 10^5^ cells were resuspended in 1 mL of chondrogenic differentiation medium and transferred into 15 mL conical tubes to allow spontaneous cell aggregation. Cell pellets were maintained under chondrogenic conditions for the entire differentiation period, with medium replacement performed three times per week Control samples consisted of cells maintained in standard culture medium without differentiation supplements. At the end of the 24–day induction period, cell aggregates were embedded in optimal cutting temperature (OCT) compound, frozen, and sectioned using a cryostat. Chondrogenic differentiation was assessed by immunofluorescence detection of aggrecan (ACAN), a cartilage-specific extracellular matrix protein. Sections were incubated with a mouse monoclonal anti-aggrecan antibody (Clone 969D4D11 Invitrogen; dilution 1:25), followed by staining with a rhodamine-conjugated isotype-specific secondary antibody (Chemicon International, Temecula, CA, USA; dilution 1:100).

### 2.4. RNA Extraction and Reverse Transcription

Total RNA was extracted from both undifferentiated and chondrogenically differentiated ADSCs using the Maxwell automated purification system (Promega, Madison, WI, USA) in combination with the RNA Blood Kit, following the manufacturer’s recommendations. RNA quantity and quality were assessed prior to downstream applications. For complementary DNA (cDNA) synthesis, 1 μg of total RNA was reverse transcribed in a final reaction volume of 20 μL. Each reaction mixture contained 8 μL of 10× reaction buffer, 4.8 μL of 25 mM MgCl_2_, 2 μL of ImpromII reverse transcriptase (Promega), 1 μL of RNase inhibitor (20 U/μL), 0.4 μL of random hexamers (250 μM), 2 μL of dNTP mix (100 mM), and nuclease-free water to reach the final volume. Reverse transcription was performed using a GeneAmp PCR System 9700 Thermal Cycler (Applied Biosystems, Foster City, CA, USA) under the following conditions: incubation at 25 °C for 5 min, extension at 42 °C for 60 min, and enzyme inactivation at 70 °C for 15 min. The resulting cDNA samples were subsequently stored at −80 °C until use in real-time PCR analyses.

### 2.5. Transcription Levels of ACAN for Chondrogenic Differentiation

The relative mRNA expression levels of ACAN and glyceraldehyde-3-phosphate dehydrogenase (GAPDH) were determined using the Chondro RT-PCR commercial kit (BioMole, Turin, Italy, cod. BM-015), following the manufacturer’s protocol. Amplification reactions were carried out in a 96-well plate with the following cycling conditions: an initial denaturation step at 95 °C for 2 min, followed by 40 cycles of 95 °C for 15 s and 60 °C for 1 min. Each kit includes a multiple-target plasmid (pDiff) containing the full-length sequences of the ACAN and GAPDH amplicons, which was employed in every PCR run as a positive control.

Normalization of target gene expression was performed using GAPDH as the endogenous reference gene. Relative changes in transcript levels between differentiated and undifferentiated ADSCs were calculated using the ΔΔCt (delta–delta Ct) method [12], and results were reported as Relative Quantification (RQ) values.

### 2.6. Transcription Levels of Pol Genes HERV-H, -K, -W, TRIM28/SETDB1 and NANOG, OCT-4, SOX-2

The transcriptional levels of *pol* genes belonging to HERV-H, HERV-K, and HERV-W, as well as those of the epigenetic regulators TRIM28 and SETDB1 and the pluripotency-associated genes NANOG, OCT4, and SOX2, were quantified by real-time PCR. Primer and probe sequences used for each target are reported in Table 1.

Real-time PCR reactions were performed using 40 ng of cDNA in a final volume of 20 μL. Each reaction mixture contained GoTaq Master Mix (2.5 U; Promega), 1.25 mmol/L MgCl_2_, 500 nM of each gene-specific primer, and 200 nM of the corresponding TaqMan probe. Amplifications were carried out in a 96-well plates using standard thermal cycling conditions consisting of an initial denaturation step at 95 °C for 10 min, followed by 45 cycles of denaturation at 95 °C for 15 s and annealing/extension at 60 °C for 1 min. All reactions were performed in technical triplicates.

Cycle threshold (Ct) values were obtained for all target genes in every analyzed sample, confirming the suitability of the experimental approach for the detection and quantification of HERV transcripts. As in our previous studies [13,14,15,16], GAPDH was used as the endogenous reference gene.

Relative transcript levels were calculated using the ΔΔCt method [11] and expressed as Relative Quantification (RQ) values.

All experimental procedures were conducted in a biosafety level 2 (BSL-2) laboratory, in compliance with the relevant biosafety guidelines [17,18].

### 2.7. Statistical Analysis

Data collection, processing, and graphical visualization were performed using Microsoft Excel. GraphPad Prism software (version 7) was employed for the statistical analyses. Differences in transcriptional levels of HERV-H-*pol*, HERV-K-*pol*, HERV-W-*pol*, TRIM28, SETDB1, NANOG, OCT4, and SOX2 between chondrogenically differentiated ADSCs and control cells were evaluated using the Wilcoxon paired-sample test. A *p*-value < 0.05 was considered indicative of statistical significance.

## 3. Results

### 3.1. Confirmation of Chondrogenic Differentiation

**Figure 1:**
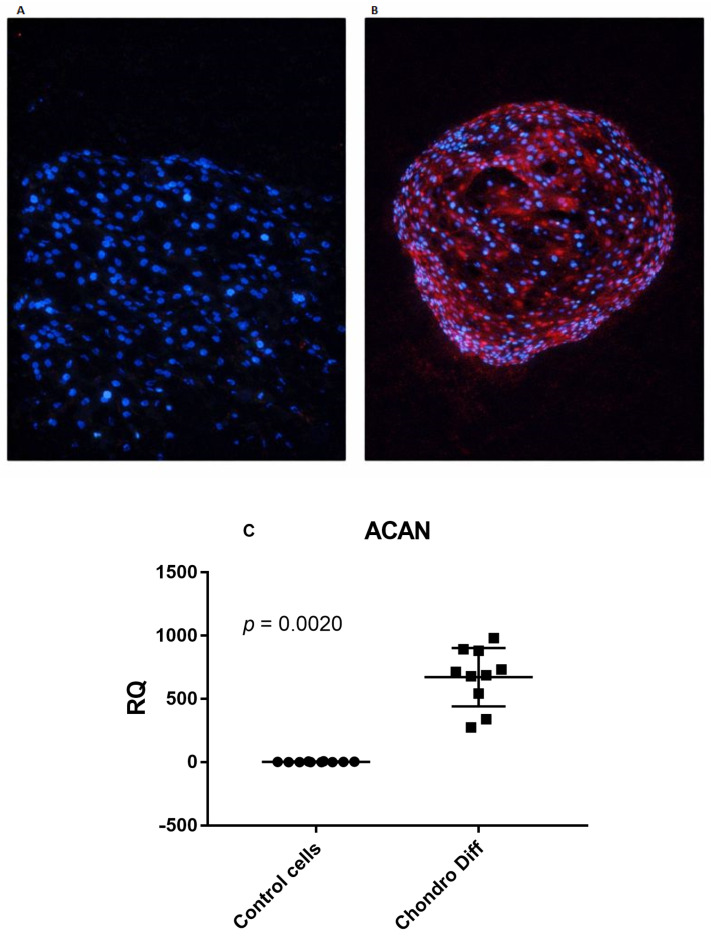
Confirmation of chondrogenic differentiation od ADSCs. (**A**) Undifferentiated ADSCs showing only nuclear DAPI staining (blue) and absence of aggrecan signal. Scale bar: 100 μm. (**B**) ADSCs after 24 days in chondrogenic induction medium, forming three-dimensional aggregates with strong aggrecan immunoreactivity (red) within the extracellular matrix. Nuclei are counterstained with DAPI (blue). Scale bar: 100 μm. (**C**) Relative quantification (RQ) of ACAN mRNA expression in control and chondrogenic differentiated ADSCs, normalized to GAPDH. Circles and squares show the individual measurements; horizontal lines represent mean values and error bars indicate standard deviation (SD).

Chondrogenic differentiation of ADSCs was confirmed by morphological analysis. After 24 days of culture in chondrogenic induction medium, ADSCs formed compact three-dimensional aggregates typical of chondrogenic lineage commitment. Immunofluorescence staining for aggrecan revealed clear differences between the two conditions: undifferentiated control cells showed no detectable aggrecan signal (Figure 1A), whereas differentiated aggregates exhibited strong extracellular matrix positivity (Figure 1B).

In parallel, quantitative real-time PCR analysis demonstrated a marked upregulation of ACAN mRNA expression in differentiated ADSCs compared with undifferentiated controls. Using TaqMan-based real-time PCR normalized to GAPDH, ACAN expression was significantly increased following chondrogenic differentiation (*p* = 0.0020) (Figure 1C).

The mean values + SD were: ACAN: 2.55 ± 2.75 in control cells and 672.40 ± 230.92 after adipogenic differentiation.

Together, these morphological, immunofluorescence, and transcriptional findings confirm the effective chondrogenic differentiation of ADSCs under the applied experimental conditions.

### 3.2. Expression of HERV-H, HERV-K and HERV-W in Control and Chondrogenic Differentiated ADSCs

**Figure 2:**
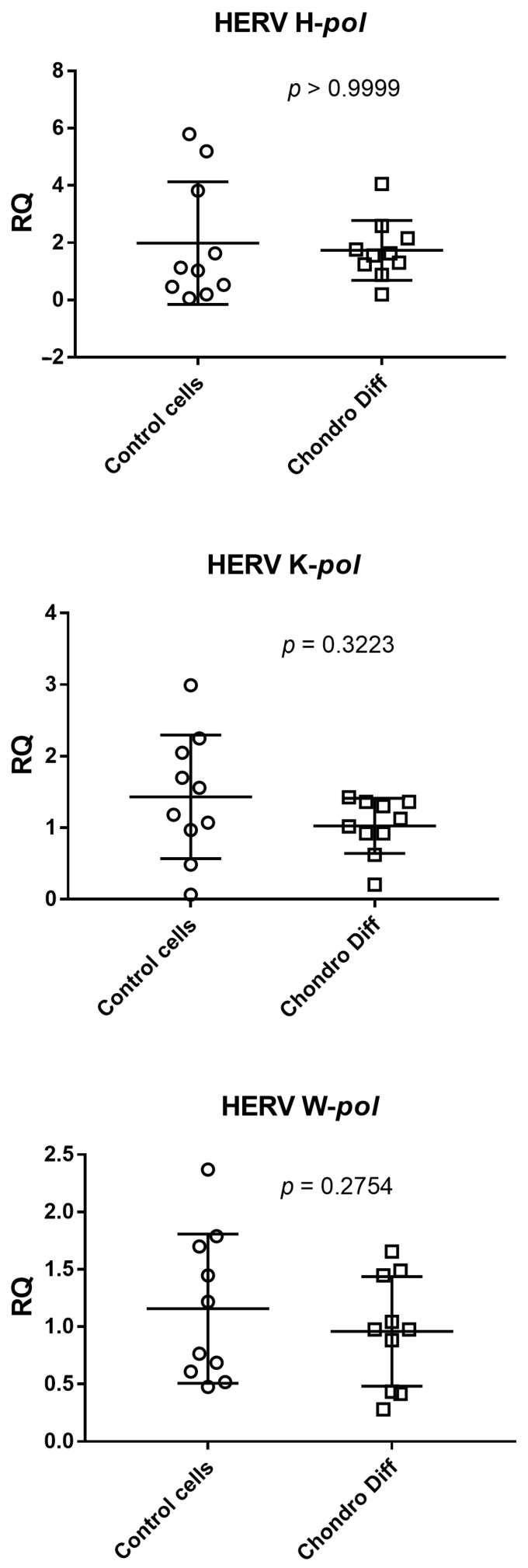
Expression of HERV-H, HERV-K and HERV-W in control and chondrogenic differentiated ADSCs. RQ: Relative Quantification; Control cells: cells not differentiated; Chondro Diff: chondrogenic differentiated cells; circles and squares show the individual measurements; horizontal lines represent the mean values; error bars represent the standard deviation (SD).

Relative quantification of mRNA expression of *pol* genes of HERV-H, HERV-K and HERV-W was evaluated in ten control and ten chondrogenic differentiated ADSCs. No statistically significant differences were observed between the two groups (Figure 2).

The mean values + standard deviations were: HERV H-*pol:* 1.96 ± 2.14 in control cells and 1.74 ± 1.05 after chondrogenic differentiation; HERV K-*pol*: 1.43 ± 0.86 in control cells and 1.03 ± 0.38 after chondrogenic differentiation; HERV W-*pol*: 1.16 ± 0.65 in control cells and 0.96 ± 0.48 after chondrogenic differentiation.

Analyzing the means of the determinations, HERV-K and HERV-W showed reduced values after differentiation, while HERV-H remained unchanged.

### 3.3. Expression of SETDB1 and TRIM28 in Control and Chondrogenic Differentiated ADSCs

**Figure 3:**
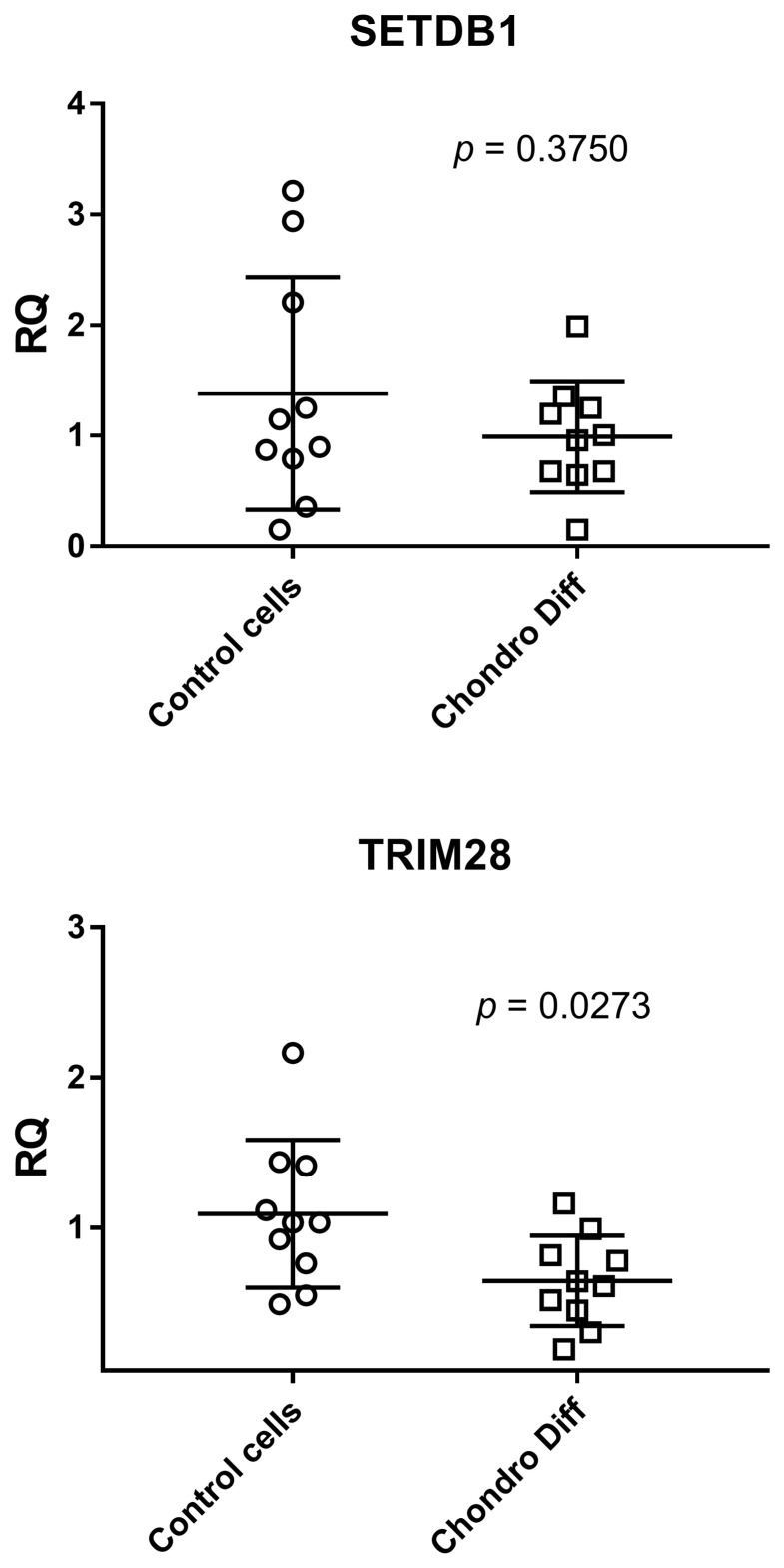
Expression of SETDB1 and TRIM28 in control and chondrogenic differentiated ADSCs. RQ: Relative Quantification; Control cells: cells not differentiated; Chondro Diff: chondrogenic differentiated cells; circles and squares show the individual measurements; horizontal lines represent the mean values; error bars represent the standard deviation (SD).

Relative quantification of mRNA expression of SETDB1 and TRIM28 was evaluated in ten control and ten chondrogenic differentiated ADSCs. ADSCs showed a statistically significant reduced expression of TRIM28 after differentiation (*p* = 0.0273), while SETDB1 showed only a reduction trend without reaching the statistical significance between the two groups (Figure 3).

The mean values + standard deviations were: SETDB1: 1.38 ± 1.05 in control cells and 0.99 ± 0.50 after chondrogenic differentiation; TRIM28: 1.09 ± 0.49 in control cells and 0.65 ± 0.30 after chondrogenic differentiation.

Analyzing the means of the determinations, both SETDB1 and TRIM28 showed reduced values after chondrogenic differentiation.

### 3.4. Correlation Analysis Between HERV Expression and Epigenetic Regulators

Correlation analyses performed in undifferentiated ADSCs did not reveal any significant associations between the expression of HERV-H, HERV-K, or HERV-W and the epigenetic regulators TRIM28 or SETDB1 (all *p* > 0.05).

In contrast, differentiated ADSCs displayed a distinct correlation pattern. While no significant correlations were observed for HERV-H or HERV-K, HERV-W expression showed a significant negative correlation with TRIM28 (r = −0.7295, *p* = 0.0204) and a significant positive correlation with SETDB1 (r = 0.7652, *p* = 0.0124).

These results suggest that HERV-W is differentially regulated by TRIM28 and SETDB1 during chondrogenic differentiation, whereas HERV-H and HERV-K do not appear to exhibit coordinated regulation with these epigenetic factors under the conditions tested (Table 2).

### 3.5. Expression of NANOG, OCT4 and SOX2 in Control and Chondrogenic Differentiated ADSC

**Figure 4:**
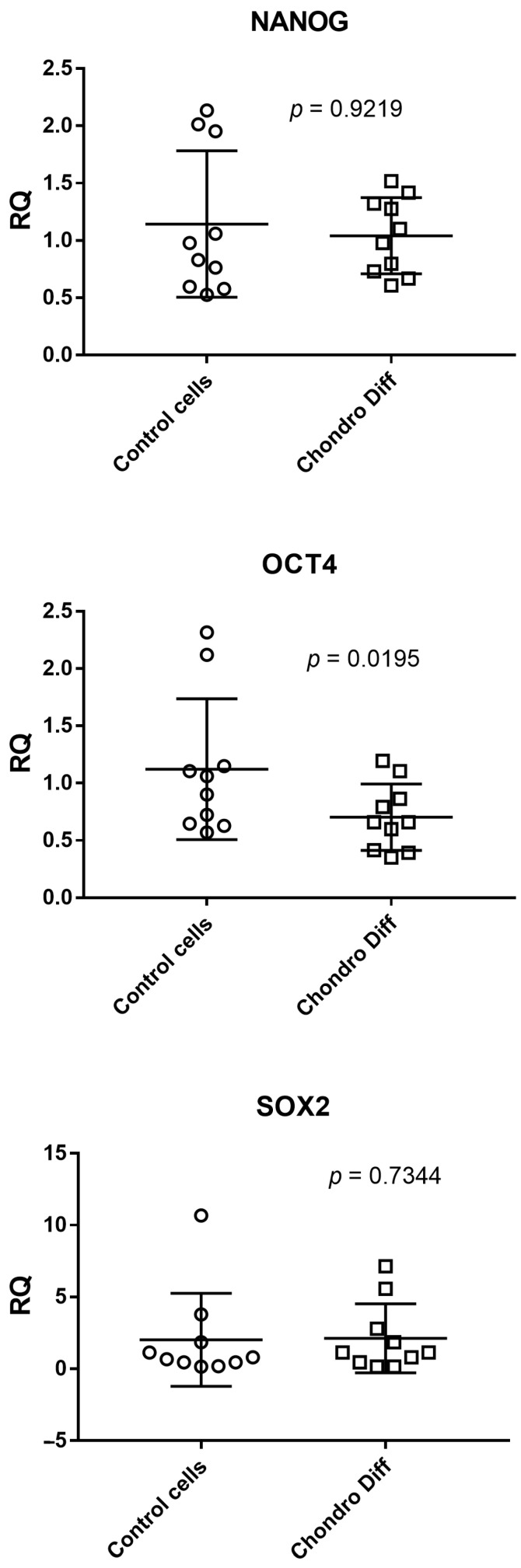
Expression of NANOG, OCT4 and SOX2 in control and chondrogenic differentiated ADSCs. RQ: Relative Quantification; Control cells: cells not differentiated; Chondro Diff: chondrogenic differentiated cells; circles and squares show the individual measurements; horizontal lines represent the mean values; error bars represent the standard deviation (SD).

Relative quantification of mRNA expression of NANOG, OCT4 and SOX2 was evaluated in ten control and ten chondrogenic differentiated ADSCs. OCT4 showed a significant reduced expression in chondrogenic differentiated ADSCs than in control cells (*p* = 0.0195), while the expression of NANOG and SOX2 was not statistically different between the two groups. The mean values + standard deviations were: NANOG: 1.14 ± 0.64 in control cells and 1.04 ± 0.33 after chondrogenic differentiation; OCT4: 1.12 ± 0.62 in control cells and 0.70 ± 0.29 after chondrogenic differentiation; SOX2: 2.02 ± 3.23 in control cells and 2.12 ± 2.40 after chondrogenic differentiation (Figure 4).

Analyzing the means of the determinations, OCT4 showed a reduced value after differentiation, while for NANOG and SOX2 this trend was not visible.

## 4. Discussion

Mesenchymal stem cells (MSCs) are multipotent progenitor cells broadly distributed across various tissues of the human body, capable of differentiating into multiple specialized cell types. Their differentiation is generally influenced by specific extrinsic stimuli such as cytokines, signaling molecules, and physical factors that activate distinct intracellular pathways [19].

A wide spectrum of RNA molecules contributes to the regulation of MSC chondrogenic differentiation, including microRNAs (miRNAs), long noncoding RNAs (lncRNAs), and RNAs derived from transposable element (TE) sequences. Previous studies have identified several miRNAs: miR-130b, miR-218, miR-495, miR-30a, and miR-204 as important modulators of chondrogenic lineage commitment [20,21,22,23,24]. Unlike small ncRNAs, lncRNAs are able to fold into complex secondary and tertiary structures, enabling diverse modes of interaction with both proteins and nucleic acid targets. These molecules play essential roles in regulating gene expression during developmental and differentiation processes and are involved in a wide range of cellular and physiological pathways [25].

Among the lncRNAs associated with chondrogenesis, ZBED3-AS1 and CTA-941F9.9 have been highlighted through co-expression and target prediction analyses as potentially key regulators of this process [25]. Moreover, recent findings suggest that irisin may enhance chondrogenic differentiation by modulating critical lncRNAs such as XIST and DLX6-AS1 [26].

Transposable elements (TEs) represent a heterogeneous group of genomic sequences capable of relocating within the genome, often through replicative mechanisms. Over evolutionary time, TEs have expanded extensively through transposition and duplication events, now comprising nearly 40% of the mammalian genome. Although most human TEs are inactive remnants unable to transpose, emerging evidence indicates that these elements still play functional roles in both normal cellular physiology and disease. Notably, TE-derived sequences are highly represented within lncRNAs, suggesting that embedded TE fragments may contribute to lncRNA-mediated gene regulation, though this aspect remains relatively underexplored [27].

Human endogenous retroviruses (HERVs) are a subclass of retrotransposons alongside DNA transposons collectively referred to as transposable elements. Historically dismissed as “junk DNA,” advances in genome-wide expression and epigenetic profiling have revealed their significant functional impact on development and disease processes [9]. HERVs are now understood to actively participate in gene regulation by recruiting transcription factors, serving as alternative promoters, encoding lncRNAs, and even producing functional proteins that influence cellular activities. These roles may originate from their intrinsic viral functions or from evolutionary co-option by the host genome. Consequently, HERVs have become integral components of the genomic regulatory network essential for mammalian development and homeostasis.

Although ERVs and other TE families exhibit high transcriptional activity during early embryogenesis, their expression is typically repressed as differentiation progresses. This silencing is achieved through coevolutionary mechanisms involving host chromatin regulatory systems that restrict ERV transcription and transposition [9]. Research on the contribution of ERVs to stem cell fate determination and lineage specification is still emerging, and many aspects of their regulatory mechanisms remain unresolved.

In the present study, we observed a trend for decrease in the expression of the *pol* genes of HERV-K and HERV-W in mesenchymal stem cells undergoing chondrogenic differentiation, while HERV-H expression remained unchanged. Our previous work demonstrated a correlation between the expression of HERV-K, -W, and -H *pol* genes and the pluripotency-associated transcription factors NANOG, OCT4, and SOX2 [1]. During differentiation, OCT4 expression declined in parallel with HERV-K and HERV-W, whereas SOX2 and NANOG levels remained stable. These observations suggest that the distinct expression patterns may reflect differential regulatory kinetics of these genes during the chondrogenic process.

The differential behavior of pluripotency factors observed in our study is consistent with findings reported in other stem cell systems. Several studies have shown that OCT4, NANOG, and SOX2 do not necessarily follow identical expression dynamics during lineage commitment. In human mesenchymal stem cells, OCT4 has been reported to undergo faster and more pronounced downregulation compared with NANOG and SOX2, highlighting a differential sensitivity of these factors to early differentiation cues [28]. Moreover, pluripotency regulators operate within a complex network in which their repression occurs asynchronously through multiple transcriptional and epigenetic mechanisms [28]. Studies in pluripotent stem cells further support this model, showing that OCT4 is among the earliest factors to be silenced during commitment, whereas NANOG and SOX2 can persist for longer periods or display context-dependent modulation [29]. Although our study focused on transcriptional expression, future investigations evaluating OCT4, NANOG and SOX2 at the protein level would help clarify whether these selective transcriptional patterns translate into functional differences during chondrogenic induction. Zinc finger proteins (ZFPs) constitute a large and diverse family of transcription factors characterized by the presence of zinc finger domains (ZFDs) [30]. The DNA-binding specificity of ZFPs is defined by the amino acid composition of an α-helix within the ZFD, while additional domains mediate the recruitment of cofactors responsible for chromatin modification [30]. Host cells exploit ZFPs to recognize sequence motifs typical of ERV loci and to recruit cofactors that suppress transcription initiation. Specifically, the KRAB domain of ZFPs binds to the TRIM28–SETDB1 complex, which catalyzes DNA methylation and H3K9 trimethylation, resulting in transcriptional silencing of ERV-associated genomic regions [31]. Subsequently, the TRIM28–SETDB1 complex recruits additional chromatin regulators, including the HIRA complex and the DAXX–ATRX heterodimer, which act as histone chaperones to replace canonical H3 with the transcriptionally repressive H3.3 variant, thereby promoting chromatin compaction and transcriptional repression [32].

Although our data do not directly assess whether TRIM28 and SETDB1 regulate chondrogenic genes, their established epigenetic functions allow us to hypothesize possible mechanistic links. Both TRIM28 and SETDB1 participate in repressive chromatin complexes that mediate H3K9 trimethylation and heterochromatin formation, largely through the KRAB-ZFP pathway [30,31]. These epigenetic mechanisms contribute to transcriptional repression by modulating chromatin accessibility and histone variant incorporation, including H3.3, which plays a key role in transcriptional regulation during cellular differentiation [32]. During chondrogenic differentiation, the observed reduction in TRIM28 and SETDB1 expression may therefore contribute to a more permissive chromatin environment, potentially facilitating the activation of lineage-defining genes such as SOX9, ACAN, and type II collagen (COL2A1). Alternatively, TRIM28 and SETDB1 may influence differentiation indirectly by modulating HERV transcriptional activity, with downstream effects on nearby regulatory elements. The opposite correlations identified for HERV-W with TRIM28 and SETDB1 in differentiated ADSCs support the possibility that these epigenetic factors engage distinct regulatory pathways during lineage commitment. Further studies using chromatin immunoprecipitation or targeted epigenetic perturbation will be required to clarify whether TRIM28 and SETDB1 directly modulate chondrogenic gene promoters or enhancers [33].

Our findings confirm the involvement of this HERV–TRIM28–SETDB1 regulatory axis during chondrogenic differentiation, as evidenced by reduced TRIM28 and SETDB1 expression concomitant with the downregulation of HERV transcripts. Correlation analyses further supported this regulatory link: in differentiated ADSCs, HERV-W expression showed a significant negative correlation with TRIM28 and a significant positive correlation with SETDB1, while no correlations were observed for HERV-H or HERV-K. These results suggest that HERV-W may be more dynamically responsive to epigenetic modulation during the chondrogenic differentiation process. The rapid diversification of KRAB-ZFPs in mammals and the wide variety of ERVs across species support the hypothesis of coevolution between these regulatory proteins and their retroviral targets [30]. Despite recent insights, additional TRIM28-independent pathways contributing to ERV regulation have been identified, underscoring the complexity of host–ERV interactions. Further studies are required to fully elucidate how mammalian cells have adapted to control integrated ERVs and the extent to which these elements influence normal cellular function.

The correlation patterns observed in this study provide additional insight into the epigenetic regulation of HERVs during lineage commitment. The absence of significant associations in undifferentiated ADSCs indicates that HERV expression and epigenetic regulator levels fluctuate independently under basal conditions. However, the emergence of significant correlations exclusively for HERV-W in differentiated cells—negative with TRIM28 and positive with SETDB1—highlights a potential shift in regulatory dynamics triggered by chondrogenic induction. This selective responsiveness of HERV-W is consistent with previous evidence suggesting that different HERV families may be regulated through distinct epigenetic mechanisms. Future time-course studies including intermediate differentiation stages will be essential to clarify how the interplay between HERVs and epigenetic regulators evolves throughout the differentiation process.

From a translational perspective, these findings may have relevant implications for tissue engineering and regenerative medicine. ADSCs are widely explored for cartilage repair [2], and understanding how HERVs and their epigenetic regulators behave during chondrogenic differentiation could contribute to optimizing cell-based therapeutic strategies. The differential regulation observed for HERV-W suggests that endogenous retroelements may influence the efficiency or stability of lineage commitment, potentially affecting the quality of engineered cartilage constructs. Modulating TRIM28–SETDB1–HERV pathways could therefore represent a strategy to enhance chondrogenic induction or to reduce undesired phenotypic drift during cell expansion. Moreover, profiling HERV expression and its epigenetic regulators may help identify molecular markers predictive of differentiation potential, thereby supporting personalized approaches to regenerative medicine [1]. Future studies integrating these molecular insights into scaffold-based or cell-based cartilage regeneration paradigms may ultimately improve clinical outcomes in cartilage repair.

## 5. Conclusions

The present study provides new insight into the relationship between HERV expression, pluripotency factors, and epigenetic regulators during the chondrogenic differentiation of adipose-derived mesenchymal stem cells. Although no significant differences were observed in HERV transcription, HERV-K and HERV-W showed a tendency toward decreased expression, and a significant reduction in TRIM28 and OCT4 was detected after differentiation. Correlation analyses revealed a selective regulatory association between HERV-W and the epigenetic regulators TRIM28 and SETDB1, suggesting a family-specific modulation of retroelement activity during lineage commitment. These findings highlight the complexity of the molecular networks involved in MSC differentiation and point toward the potential value of HERV-related markers and epigenetic regulators in future studies aimed at improving tissue engineering and regenerative medicine strategies.

## Figures and Tables

**Table 1:** Primers and probes used to assess the transcription levels of *pol* genes of HERV-H, HERV-K, and HERV-W, of TRIM28 and SETDB1, of NANOG, OCT-4, SOX-2 and of GAPDH.

Name	Primer/Probe	Sequence
HERV-H *pol*	Forward	5′-TGGACTGTGCTGCCGCAA-3′
	Reverse	5′-GAAGSTCATCAATATATTGAATAAGGTGAGA-3′
	Probe	6FAM-5′-TTCAGGGACAGCCCTCGTTACTTCAGCCAAGCTC-3′-TAMRA
HERV-K *pol*	Forward	5′-CCACTGTAGAGCCTCCTAAACCC-3′
	Reverse	5′-TTGGTAGCGGCCACTGATTT-3′
	Probe	6FAM-5′-CCCACACCGGTTTTTCTGTTTTCCAAGTTAA-3′-TAMRA
HERV-W *pol*	Forward	5′-ACMTGGAYKRTYTTRCCCCAA-3′
	Reverse	5′-GTAAATCATCCACMTAYYGAAGGAYMA-3′
	Probe	6FAM-5′-TYAGGGATAGCCCYCATCTRTTTGGYCAGGCA-3′-TAMRA
TRIM28	Forward	5′-GCCTCTGTGTGAGACCTGTGTAGA-3′
	Reverse	5′-CCAGTAGAGCGCACAGTATGGT-3′
	Probe	6FAM-5′-CGCACCAGCGGGTGAAGTACACC-3′-TAMRA
SETDB1	Forward	5′-GCCGTGACTTCATAGAGGAGTATGT-3′
	Reverse	5′-GCTGGCCACTCTTGAGCAGTA-3′
	Probe	6FAM-5′-TGCCTACCCCAACCGCCCCAT-3′-TAMRA
NANOG	Forward	5′-GCCAGGATGGTCTCGATCTC-3′
	Reverse	5′-GGTGGCTCACGCCTGTAAAT-3′
	Probe	6FAM- TGACCTTGTGATCCACCCGCCTC–TAMRA
OCT-4	Forward	5′-ACCCACACTGCAGCAGATCA-3′
	Reverse	5′-CACACTCGGACCACATCCTTCT-3′
	Probe	6FAM-CCACATCGCCCAGCAGCTTGG–TAMRA
SOX-2	Forward	5′-TGCGAGCGCTGCACAT-3′
	Reverse	5′-GCAGCGTGTACTTATCCTTCTTCA-3′
	Probe	6FAM-CCGGCGGAAAACCAAGACGCT–TAMRA
GAPDH	Forward	5′-CGAGATCCCTCCAAAATCAA-3′
	Reverse	5′-TTCACACCCATGACGAACAT-3′
	Probe	6FAM-5′-TCCAACGCAAAGCAATACATGAAC-3′-TAMRA

**Table 2:** Correlation coefficients between HERV expression and the epigenetic regulators TRIM28 and SETDB1 in chondrogenically differentiated ADSCs.

Gene Pair	Correlation Coefficient (r)	*p*-Value	Interpretation
HERV-W/TRIM28	–0.7295	0.0204	Significant negative correlation
HERV-W/SETDB1	+0.7652	0.0124	Significant positive correlation
HERV-H/TRIM28	n.s.	>0.05	No significant correlation
HERV-H/SETDB1	n.s.	>0.05	No significant correlation
HERV-K/TRIM28	n.s.	>0.05	No significant correlation
HERV-K/SETDB1	n.s.	>0.05	No significant correlation

## Data Availability

The data presented in this study are available on request from the corresponding author due to privacy.

## References

[1] Daprà V., Alotto D., Casarin S., Gambarino S., Zavatto C., Dini M., Galliano I., Castagnoli C., Bergallo M. (2022). A new protocol for validation of Chondro, Adipo and Osteo differentiation kit of Cultured Adipose-Derived Stem Cells (ADSC) by real-time rt-QPCR. Tissue Cell.

[2] Klimczak A., Kozłowska U. (2016). Mesenchymal Stromal Cells and Tissue-Specific Progenitor Cells: Their Role in Tissue Homeostasis. Stem Cells Int..

[3] Cuevas-Díaz Duran R., González-Garza M.T., Cardenas-López A., Chávez-Castilla L., Cruz-Vega D.E., Moreno-Cuevas J.E. (2013). Age-related yield of adipose-derived stem cells bearing the low-affinity nerve growth factor receptor. Stem Cells Int..

[4] Dominici M., Le Blanc K., Mueller I., Slaper-Cortenbach I., Marini F., Krause D., Deans R., Keating A., Prockop D.J., Horwitz E. (2006). Minimal criteria for defining multipotent mesenchymal stromal cells. The International Society for Cellular Therapy position statement. Cytotherapy.

[5] Mareschi K., Montanari P., Rassu M., Galliano I., Daprà V., Adamini A., Castiglia S., Fagioli F., Bergallo M. (2019). Human Endogenous Retrovirus-H and K Expression in Human Mesenchymal Stem Cells as Potential Markers of Stemness. Intervirology.

[6] Grandi N., Tramontano E. (2018). Human Endogenous Retroviruses Are Ancient Acquired Elements Still Shaping Innate Immune Responses. Front. Immunol..

[7] Engel K., Wieland L., Krüger A., Volkmer I., Cynis H., Emmer A., Staege M.S. (2021). Identification of Differentially Expressed Human Endogenous Retrovirus Families in Human Leukemia and Lymphoma Cell Lines and Stem Cells. Front. Oncol..

[8] Xue B., Sechi L.A., Kelvin D.J. (2020). Human Endogenous Retrovirus K (HML-2) in Health and Disease. Front Microbiol..

[9] Xiang Y., Liang H. (2021). The Regulation and Functions of Endogenous Retrovirus in Embryo Development and Stem Cell Differentiation. Stem Cells Int..

[10] Coleman S.R., Saboeiro A.P. (2007). Fat grafting to the breast revisited: Safety and efficacy. Plast. Reconstr. Surg..

[11] Miltenyi Biotec (2013). StemMACS™ ChondroDiff Media: Instructions for Use.

[12] Livak K.J., Schmittgen T.D. (2001). Analysis of relative gene expression data using real-time quantitative PCR and the 2^−ΔΔCT^ Method. Methods.

[13] Tovo P.A., Galliano I., Parodi E., Calvi C., Gambarino S., Licciardi F., Dini M., Montanari P., Branca M., Ramenghi U. (2023). Children with Chronic Immune Thrombocytopenia Exhibit High Expression of Human Endogenous Retroviruses TRIM28 and SETDB1. Genes.

[14] Tovo P.A., Ribaldone D.G., Galliano I., Caviglia G.P., Dini M., Veglio V., Calvi C., Montanari P., Pitoni D., Frara S. (2024). Enhanced Transcription of Human Endogenous Retroviruses and TRIM28 Downregulation in Patients with Inflammatory Bowel Disease. Viruses.

[15] Simula E.R., Jasemi S., Cossu D., Fais M., Cossu I., Chessa V., Canu M., Sechi L.A. (2025). Human Endogenous Retroviruses as Novel Therapeutic Targets in Neurodegenerative Disorders. Vaccines.

[16] Rowe H.M., Trono D. (2011). Dynamic Control of Endogenous Retroviruses during Development. Virology.

[17] National Institutes of Health (NIH) Office of Science Policy Biosafety and Biosecurity Policy. https://osp.od.nih.gov/policies/biosafety-and-biosecurity-policy/.

[18] NIH (2021). NIH Laboratory Biosafety Guidance Related to Coronavirus Disease (COVID-19): Interim Guidance. https://www.who.int/publications/i/item/WHO-WPE-GIH-2021.1.

[19] Zhou J.Q., Wan H.Y., Wang Z.X., Jiang N. (2023). Stimulating factors for regulation of osteogenic and chondrogenic differentiation of mesenchymal stem cells. World J. Stem Cells.

[20] Zhang P., Gao G., Zhou Z., He X. (2021). MicroRNA-130b downregulation potentiates chondrogenic differentiation of bone marrow mesenchymal stem cells by targeting SOX9. Braz. J. Med. Biol. Res..

[21] Chen S., Xu Z., Shao J., Fu P., Wu H. (2019). MicroRNA-218 promotes early chondrogenesis of mesenchymal stem cells and inhibits later chondrocyte maturation. BMC Biotechnol..

[22] Lee S., Yoon D.S., Paik S., Lee K.M., Jang Y., Lee J.W. (2014). MicroRNA-495 inhibits chondrogenic differentiation in human mesenchymal stem cells by targeting Sox9. Stem Cells Dev..

[23] Tian Y., Guo R., Shi B., Chen L., Yang L., Fu Q. (2016). MicroRNA-30a promotes chondrogenic differentiation of mesenchymal stem cells through inhibiting Delta-like 4 expression. Life Sci..

[24] Dalle Carbonare L., Bertacco J., Minoia A., Cominacini M., Bhandary L., Elia R., Gambaro G., Mottes M., Valenti M.T. (2022). Modulation of miR-204 expression during chondrogenesis. Int. J. Mol. Sci..

[25] Wang L., Li Z., Li Z., Yu B., Wang Y. (2015). Long noncoding RNAs expression signatures in chondrogenic differentiation of human bone marrow mesenchymal stem cells. Biochem. Biophys. Res. Commun..

[26] Chen Y., Sha W., Zhang Y., Kou W., Yang L., Guo R., Li C., Zhao J., Wang Z. (2024). Irisin-regulated lncRNAs and their potential regulatory functions in chondrogenic differentiation of human mesenchymal stem cells. Open Med..

[27] Babarinde I.A., Ma G., Li Y., Deng B., Luo Z., Liu H., Abdul M.M., Ward C., Chen M., Fu X. (2021). Transposable element sequence fragments incorporated into coding and noncoding transcripts modulate the transcriptome of human pluripotent stem cells. Nucleic Acids Res..

[28] Greco S.J., Liu K., Rameshwar P. (2007). Functional similarities among genes regulated by OCT4 in human mesenchymal and embryonic stem cells. Stem Cells.

[29] Brambrink T., Foreman R., Welstead G.G., Lengner C.J., Wernig M., Suh H., Jaenisch R. (2008). Sequential expression of pluripotency markers during direct reprogramming of somatic cells. Cell.

[30] Yang P., Wang Y., Macfarlan T.S. (2017). The role of KRAB-ZFPs in transposable element repression and mammalian evolution. Trends Genet..

[31] Turelli P., Castro-Diaz N., Marzetta F., Kapopoulou A., Raclot C., Duc J., Tieng V., Quenneville S., Trono D. (2014). Interplay of TRIM28 and DNA methylation in controlling human endogenous retroelements. Genome Res..

[32] Shi L., Wen H., Shi X. (2017). The histone variant H3.3 in transcriptional regulation and human disease. J. Mol. Biol..

[33] Gautam P., Yu T., Loh Y.H. (2017). Regulation of ERVs in pluripotent stem cells and reprogramming. Curr. Opin. Genet. Dev..

